# Monomethyl fumarate promotes Nrf2-dependent neuroprotection in retinal ischemia-reperfusion

**DOI:** 10.1186/s12974-015-0452-z

**Published:** 2015-12-21

**Authors:** Hongkwan Cho, Matthew J. Hartsock, Zhenhua Xu, Meihua He, Elia J. Duh

**Affiliations:** Department of Ophthalmology, Johns Hopkins University School of Medicine, 400 N. Broadway, Baltimore, MD 21287 USA

**Keywords:** Neurodegeneration, Retina, Ischemia-reperfusion, Fumaric acid esters/monomethyl fumarate

## Abstract

**Background:**

Retinal ischemia results in neuronal degeneration and contributes to the pathogenesis of multiple blinding diseases. Recently, the fumaric acid ester dimethyl fumarate (DMF) has been FDA-approved for the treatment of multiple sclerosis, based on its neuroprotective and anti-inflammatory effects. Its potential role as a neuroprotective agent for retinal diseases has received little attention. In addition, DMF’s mode of action remains elusive, although studies have suggested nuclear factor erythroid 2-related factor 2 (Nrf2) activation as an important mechanism. Here we investigated the neuroprotective role of monomethyl fumarate (MMF), the biologically active metabolite of DMF, in retinal ischemia-reperfusion (I/R) injury, and examined the role of Nrf2 in mediating MMF action.

**Methods:**

Wild-type C57BL/6J and Nrf2 knockout (KO) mice were subjected to 90 min of retinal ischemia followed by reperfusion. Mice received daily intraperitoneal injection of MMF. Inflammatory gene expression was measured using quantitative reverse transcription PCR (qRT-PCR) at 48 h after I/R injury. Seven days after I/R, qRT-PCR for Nrf2 target gene expression, immunostaining for Müller cell gliosis and cell loss in the ganglion cell layer (GCL), and electroretinography for retinal function were performed.

**Results:**

The results of this study confirmed that MMF reduces retinal neurodegeneration in an Nrf2-dependent manner. MMF treatment significantly increased the expression of Nrf2-regulated antioxidative genes, suppressed inflammatory gene expression, reduced Müller cell gliosis, decreased neuronal cell loss in the GCL, and improved retinal function measured by electroretinogram (ERG) after retinal I/R injury in wild-type mice. Importantly, these MMF-mediated beneficial effects were not observed in Nrf2 KO mice.

**Conclusions:**

These results indicate that fumaric acid esters (FAEs) exert a neuronal protective function in the retinal I/R model and further validate Nrf2 modulation as a major mode of action of FAEs. This suggests that DMF and FAEs could be a potential therapeutic agent for activation of the Nrf2 pathway in retinal and possibly systemic diseases.

**Electronic supplementary material:**

The online version of this article (doi:10.1186/s12974-015-0452-z) contains supplementary material, which is available to authorized users.

## Background

Retinal ischemia contributes to visual impairment and is critically involved in the pathogenesis of several diseases, including acute angle-closure glaucoma, retinal vascular occlusions, age-related macular degeneration, and diabetic retinopathy [1, 2]. Currently, there is no therapy for retinal ischemia.

Among the most widely used models to study the molecular mechanisms and test potential therapeutic approaches for retinal ischemia are rodent models of ischemia-reperfusion (I/R), in which intraocular pressure is acutely elevated above the systolic pressure for a specified period of time followed by reperfusion. Reperfusion of the retina after ischemia results in oxidative stress that is marked by the production of reactive oxygen species (ROS) and subsequent inflammatory responses [3–5], resulting in neurodegeneration manifested by the loss of retinal ganglion cells (RGC) and a significant reduction of b wave amplitude in the electroretinogram (ERG) [6, 7].

Recently, it has been shown that fumaric acid esters (FAEs) enhance survival of various central nervous system (CNS) cell types in response to oxidative insults in vitro [8–12], improve outcomes in animal models of Huntington’s disease and experimental autoimmune encephalomyelitis [8, 13], and activate the nuclear factor erythroid 2-related factor 2 (Nrf2) pathway and upregulate Nrf2-dependent antioxidant genes and proteins in the brain [8, 9]. This suggests that Nrf2 activation may be an important pathway mediating the effects of FAEs. Importantly, the fumaric acid ester dimethyl fumarate (DMF; Tecfidera/BG-12) has recently been approved by the FDA for the treatment of multiple sclerosis based on its neuroprotective and anti-inflammatory effects. Once ingested, DMF is rapidly and extensively metabolized in the GI tract by esterases into monomethyl fumarate (MMF), which is the biologically and pharmacologically active metabolite of DMF [14, 15]. Despite numerous studies showing protective effects of FAEs in CNS disease models [16, 17], little information is available on therapeutic effects of FAEs on retinal conditions.

Nrf2 is a transcription factor known to play a major cytoprotective role against endogenous and exogenous stresses and serves as one of the most important cellular pathways in protection against oxidative stress [18, 19]. Under normal physiological conditions, most Nrf2 are sequestered in the cytoplasm through interactions with its inhibitor, Keap1, which also directs Nrf2 toward ubiquitination and subsequent proteosomal degradation. Upon exposure to various endogenous or exogenous stress-inducing agents such as reactive oxygen species, Nrf2 dissociates from Keap1 and translocates to the nucleus, where it mediates the activations of an array of cytoprotective and antioxidant genes via binding to the antioxidant response element [19]. This mode of regulation is particularly amenable to pharmacological modulation, enabling Nrf2 activation by multiple drugs known to induce the Nrf2 pathway [20]. Both DMF and MMF have been shown to activate the Nrf2 pathway [8, 9, 21]; however, the importance of Nrf2 activation in their therapeutic effects remains to be established.

Here we tested the hypothesis that MMF exerts neuronal protection in the retina after I/R injury via the Nrf2 pathway. Our data show that MMF treatment induces upregulation of Nrf2 target genes, suppresses inflammatory gene expression, mitigates reactive Müller cell gliosis, inhibits I/R-induced neuronal cell loss, and partially restores electrophysiological function of the retina after I/R injury in an Nrf2-dependent manner. Together, our findings suggest that FAEs are a promising neuroprotective agent for retinal I/R and might be beneficial for other retinal and systemic conditions, especially those in which Nrf2 activation is desirable.

## Methods

### Animals

Animal studies were approved by the Institutional Animal Care and Use Committee of the Johns Hopkins University School of Medicine. All procedures involving animals were conducted in accordance with the Association for Research in Vision and Ophthalmology Statement for the Use of Animals in Ophthalmic and Vision Research. *Nrf2* knockout (KO) mice backcrossed into C57BL/6 J [2, 22] and wild-type C57BL/6 J mice (Jackson Laboratory) were used for all experiments. The mice were maintained under standard conditions on a 12-h light to dark cycle with ad libitum access to food and water. The animals were randomly assigned into each experimental group in equal numbers. Due to attrition (death during I/R or ERG procedures, or formation of severe cataract after I/R) not all groups contain the same number of animals.

### Mouse model of retinal ischemia-reperfusion and monomethyl fumarate treatment

Retinal ischemia was induced as described previously [2]. Mice were anesthetized with a cocktail of 100 mg/kg ketamine, 10 mg/kg xylazine, and 3 mg/kg acepromazine via intraperitoneal (i.p.) injection. The anterior chamber of one eye was cannulated with a 30-gauge needle attached to a line infusing 0.9 % sterile saline. The intraocular pressure (IOP) was raised to 90 mmHg by elevating the saline reservoir. The retina was monitored for blanching, which indicates retina ischemia. After 90 min of ischemia, the needle was withdrawn from the eye and the IOP was restored. The untreated contralateral eye of the same mouse was used as a control. Mice were treated with intraperitoneal injections of 50 mg/kg of MMF (dissolved in phosphate-buffered saline (PBS), Sigma Aldrich, St. Louis, MO) or PBS at 2 days, 1 day, and 0 day before I/R. After I/R, mice were treated daily with MMF until sacrificed at designated time points. The expression of antioxidant genes, Müller cell gliosis, and neuronal cell death in the ganglion cell layer (GCL) and ERG were all measured at 7 days to allow sufficient time to develop I/R-induced deficiencies. However, the inflammatory gene expression was measured at 48 h, as I/R-induced upregulation of inflammatory gene expression peaks in the first 48 h.

### Quantitative Reverse Transcription PCR

Total RNA from the retina was isolated using RNeasy mini kit (Qiagen, Valencia, CA) with a DNase (Qiagen, Valencia, CA) treatment. Single-stranded cDNA was synthesized using MMLV Reverse Transcriptase (Invitrogen). Quantitative reverse transcription PCR (qRT-PCR) was performed using the QuantiTect SYBR Green PCR Kit (Qiagen) with the StepOnePlus real-time PCR system (Applied Biosystems). The qRT-PCR primers used in this study to analyze the expression of Nrf2 target genes and inflammatory mediators are listed in Table 1. The roles of each of these genes are explained in Table S1 (Additional file 1: Table S1). The housekeeping gene Cyclophilin A was used for normalization. The fold difference between levels of transcripts was calculated using the ΔΔCT method.

**Table 1:** qRT-PCR primers used in this study

Gene	Sense primer (5′-3′)	Antisense primer (5′-3′)
*NQO1*	CAGCTCACCGAGAGCCTAGT	ACCACCTCCCATCCTTTCTT
*HO-1*	ATGACACCAAGGACCAGAGC	GTGTAAGGACCCATCGGAGA
*Prdx1*	AATGCAAAAATTGGGTATCCTGC	CGTGGGACACACAAAAGTAAAGT
*Txnrd1*	AACTTTCAGAAGGGCCAGGT	GTAGACAGGGGCGAAGACTG
*Gstm1*	AGTGGGTGGGAAAGGGTCATTACA	TAGCCAAGGC TTCTGCTGGTACTT
*IL-1β*	GAAATGCCACCTTTTGACAGTG	TGGATGCTCTCATCAGGACAG
*ICAM-1*	ACACTATGTGGACTGGCAGTGGTT	TGAGGCTCGATTGTTCAGCTGCTA
*CCL12*	ATTTCCACACTTCTATGCCTCCT	ATCCAGTATGGTCCTGAAGATCA
*CCL2*	CATCCACGTGTTGGCTCA	TCTGGACCCATTCCTTCTTG
*CCL7*	GCTGCTTTCAGCATCCAAGTG	CCAGGGACACCGACTACTG
*Cyclophilin A*	GAGCTGTTTGCAGACAAAGTT	CCCTGGCACATGAATCCTGG

### Immunohistochemistry

Following enucleation, eyes were immediately fixed in 10 % buffered formalin overnight at room temperature (RT). The fixed eyes were dehydrated with a series of ethanol washes, embedded in paraffin, and cross-sectioned (5 μm). Sections were subjected to heat-induced antigen retrieval in Target Retrieval Solution (Dako, Carpinteria, CA). Slides were blocked in 5 % goat serum in PBST (0.1 % Triton-X in PBS) for 1 h at RT and then incubated with rabbit anti-glial fibrillary acidic protein (GFAP) (1:1000) (Abcam, Cambridge, MA) for 16 h at 4 °C. After washing, the sections were incubated with goat anti-rabbit antibody (1:500) conjugated with Alexa fluor 488 (Life Technologies, Grand Island, NY) for 1 h at RT. Sections were then cover-slipped using Prolong Gold Antifade Mountant with DAPI (Life Technologies, Grand Island, NY) for nuclear staining. Retina cross-section images containing all retinal layers were captured in a blinded manner using a confocal microscope (Zeiss LSM 510, Carl Zeiss, Thornwood, NY) with a ×20 objective lens. From each eye, three non-overlapping fields from central- and mid-periphery, including all layers of the retina, were used for analysis. For each field, ten serial confocal images were taken at 1 μm intervals and then merged to produce well-focused images with maximum signal. Quantitation of immunostaining was performed as described previously [23]. Briefly, images were converted to 8-bit in ImageJ software (NIH), and an intensity threshold for immunofluorescence was set so that only GFAP-positive areas were highlighted. Results are expressed as the percentage of positive immunofluorescence area per field of retina. Investigators were masked to the groups during analysis.

### Retina whole-mount immunostaining

In order to assess surviving neurons within the GCL, retinal whole-mount staining was performed as previously described [24]. Briefly, mouse eyes were enucleated and fixed in 4 % paraformaldehyde (PFA) in PBS for 30 min at RT. The corneas and lenses were then removed, and eyecups were fixed in 4 % PFA for an additional 10 min before each retina was carefully dissected from its eyecup. Retinas were incubated for 1 hour at RT in a blocking solution containing 10 % normal goat serum in PBST (0.3 % Triton-X in PBS), followed by labeling with anti-NeuN antibody (1:600, Millipore, Billerica, MA) at 4 °C overnight. The retinas were then extensively washed in PBST for 3 h with changes of PBST every 30 min, followed by labeling with anti-mouse IgG conjugated with Alexa Fluor 488 antibody (Invitrogen, Carlsbad, CA) at 4 °C overnight. After washing with PBST, the retinas were flat-mounted on slides in Fluoromount-G (Electron Microscopy Sciences, Hatfield, PA). The survival of neuronal cells in ganglion cell layer was analyzed as previously described [25]. Retina whole-mount images were taken using a confocal microscope (Zeiss LSM 510, Carl Zeiss, Thornwood, NY) with a ×20 objective lens. Eight random fields in the retinal mid-periphery were selected from each eye. For each field, five serial z-stacked images at 1-μm intervals in ganglion cell layer were taken and then merged to produce well-focused images with maximum signal. NeuN-positive cells in GCL were manually counted in a masked fashion using the Count Tool in Adobe Photoshop CS6 (Adobe Systems Inc., San Jose, CA).

### Flash scotopic electroretinogram

All procedures were done under dim red light. Before electroretinogram (ERG) analysis, mice were dark adapted overnight. Mice were anesthetized with ketamine (100 mg/kg) and xylazine (10 mg/kg), and the pupils were dilated with a drop of 1 % Tropicamide on the corneal surface before they were placed in the Bigshot™ LED Ganzfeld stimulator (LKC Technologies, Gaithersburg, MD) on the electroretinogram system (UTAS Visual Diagnostic System, LKC Technologies, Gaithersburg, MD). Flash ERG was measured using gold wire corneal electrodes, with a forehead reference electrode and a ground electrode near the tail. Electrodes were connected to the Universal DC Biomedical Amplifier (UBS-4204, LKC Technologies, Gaithersburg, MD), and bands were filtered from 0.3 to 500 Hz. Data were recorded and analyzed using EM for Windows (Ver. 9.0.0, LKC Technologies, Gaithersburg, MD) in a blinded manner. Scotopic ERG waveforms were measured at flash intensities of −30, −20, −10, 0, 5, 10, and 15 dB, and the intensities were provided electronically by the Bigshot™ LED Ganzfeld stimulator. Ten waveforms at flash intensities of −30, −20, and −10 dB, and five waveforms at 0, 5, 10, and 15 dB were recorded. The values from each intensity were averaged in EM for Windows software (Ver. 9.0.0, LKC Technologies, Gaithersburg, MD) before analysis. To prevent the loss of dark adaptation and allow rod recovery between consecutive flashes, timing between flash intensity delivery was varied from 2 s at the two lower stimulus intensities (−30 and −20 dB) to 30 s at −10 dB and up to 60 s at the next four higher stimulus intensities (0, 5, 10, and 15 dB). The a wave was measured from the pre-stimulus baseline to the nadir of the initial corneal negative deflection; b wave was measured from the nadir of the a wave to the apex of the corneal positive wave and not up to the apex of oscillating potentials, which can exceed the b wave apex (Fig. 5d).

### Statistical analysis

All bar graphs represent mean ± SEM. Statistical analysis was performed using Student’s *t* test or one-way ANOVA with a Bonferroni correction where appropriate. *p* values < 0.05 were considered statistically significant. Statistical analyses were performed using Statplus (Analystsoft Inc, Walnut, CA).

## Results

### MMF upregulates the expression of antioxidant genes in the retina via an Nrf2-dependent pathway

**Fig. 1:**
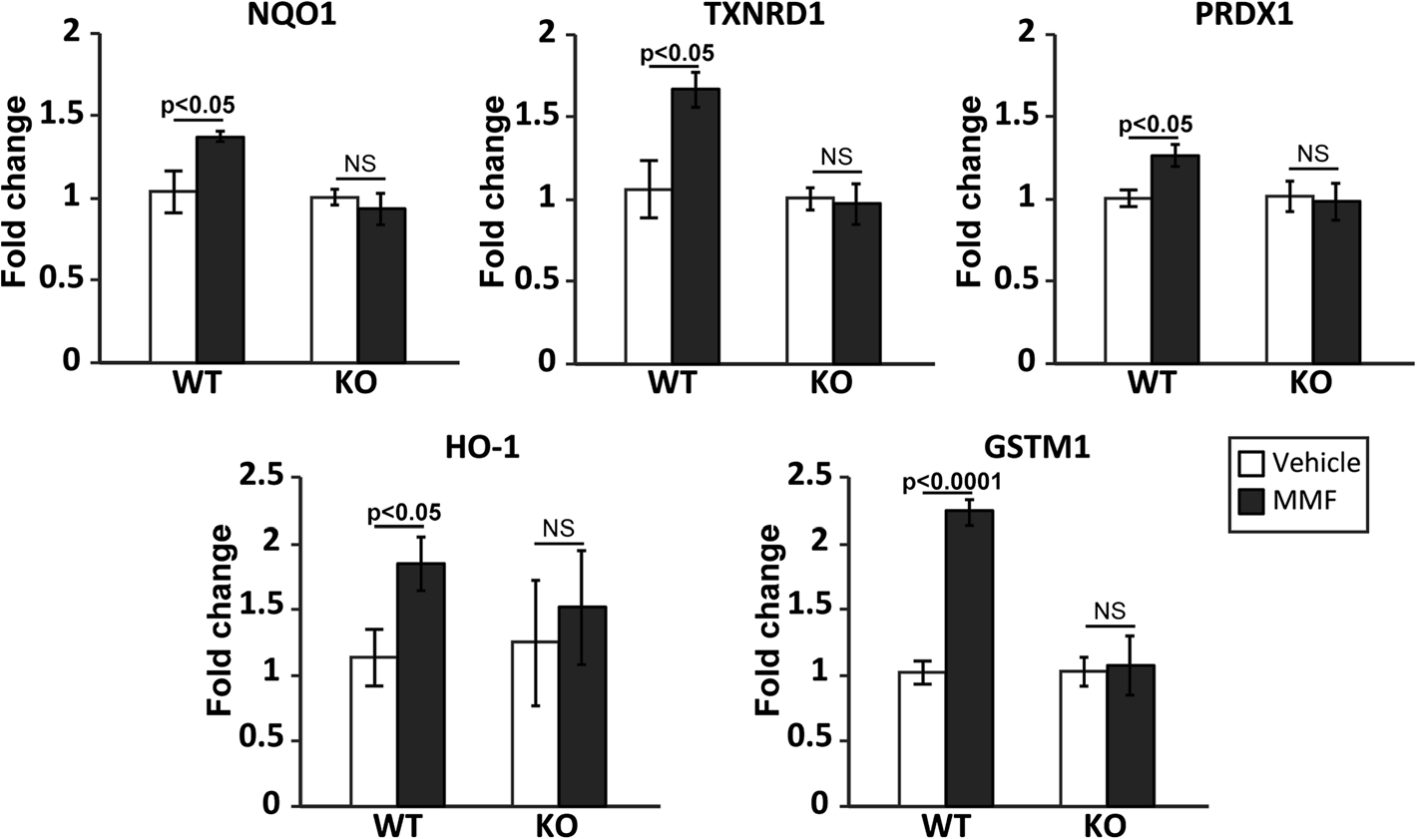
MMF-treated mice exhibit significantly increased Nrf2 target gene expression in I/R retinas in wild-type, but not Nrf2 knockout, mice. qRT-PCR analysis of Nrf2 target genes was performed on retinas harvested 7 days after reperfusion. *n* = 5-6 mice/group

Fumaric acid esters, including MMF, induce upregulation of antioxidant Nrf2 target genes in vitro [9] and exert neuroprotective effects in vivo [8]. In order to test if MMF induces mRNA expression of antioxidative enzymes [26-28] that are known to be targets of Nrf2 transcriptional activation in retinal tissue, MMF was administered daily via i.p. injections at a dose of 50 mg/kg beginning 2 days before I/R injury until sacrificed. As demonstrated in Fig. 1, MMF significantly increased Nrf2 target gene expression in I/R retinas in WT mice. Importantly, MMF did not increase the expression of these target genes in Nrf2 KO mice, confirming that this MMF effect was Nrf2-specific.

### MMF treatment suppresses inflammatory gene expression in the retina following ischemia-reperfusion

**Fig. 2:**
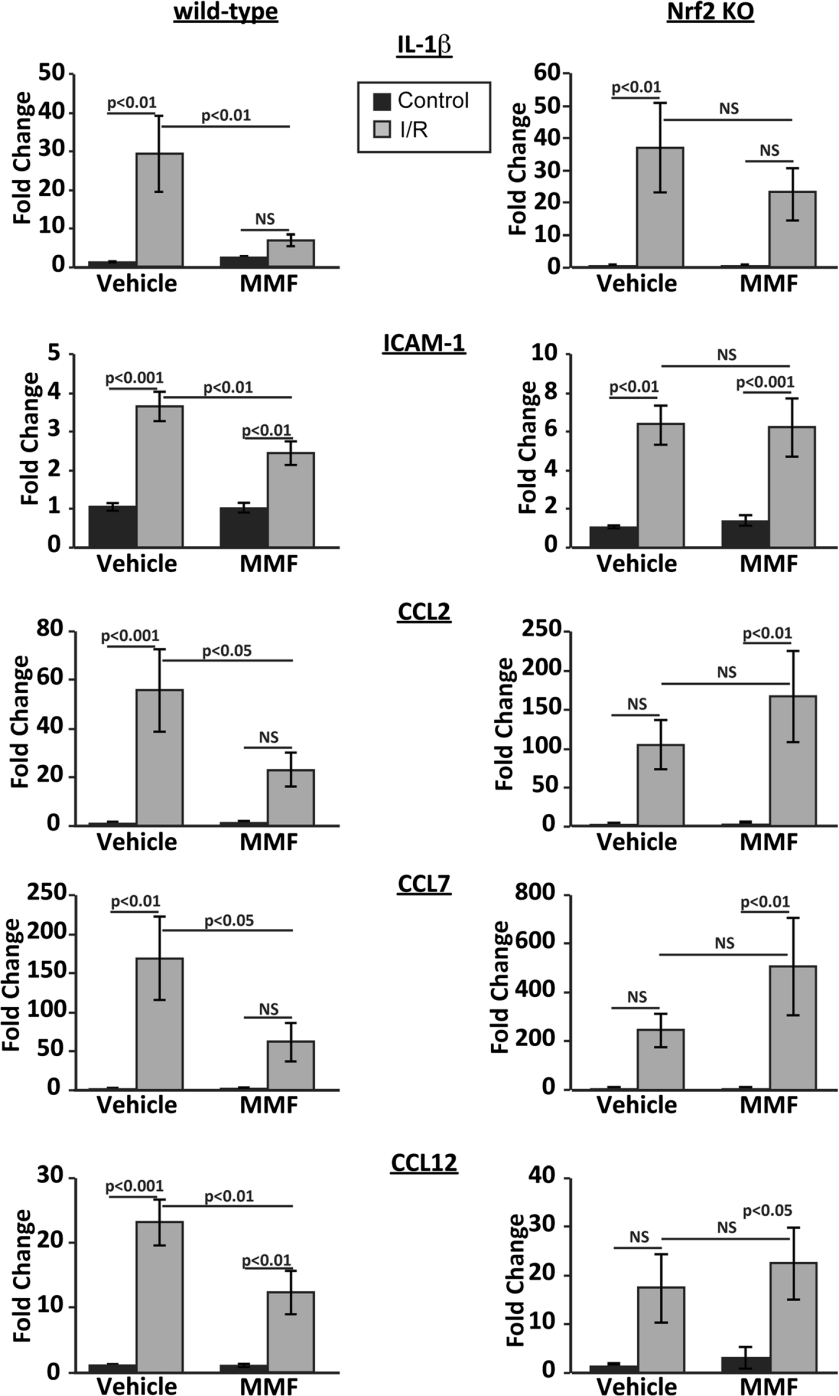
MMF treatment significantly suppresses inflammatory gene expression following I/R in wild-type, but not Nrf2 knockout, mice. qRT-PCR analysis of inflammatory mediators was performed on retinas harvested 48 h after reperfusion. *n* = 9-10 mice/group

The absence of oxygen by prolonged ischemia creates a condition in which the restoration of oxygen levels by reperfusion results in a surge in the generation of reactive oxygen species, leading to inflammation [29]. DMF and MMF have been demonstrated to suppress inflammatory cytokine production in the CNS and systemic settings [30]. IL-1β, CCL2, and ICAM-1 are classical inflammatory genes that are known to be significantly increased in I/R [31–33]. CCL2, in particular, is involved in the inflammatory responses via recruitment of monocytes, memory T cells, and dendritic cells to sites of injury [34–37]. In addition, CCL2 as well as CCL7 and CCL12 is the ligand for the CCR2 receptor, which is known to play an important role in inflammatory diseases of the CNS [38, 39]. As shown in Fig. 2, MMF treatment significantly suppressed I/R-induced upregulation of these inflammatory mediators at 48 h after reperfusion in wild-type, but not Nrf2 knockout, mice, implicating that MMF exerts anti-inflammatory effects in the retina in an Nrf2-dependent manner.

### MMF treatment mitigates Müller cell gliosis induced by ischemia-reperfusion in the retina

**Fig. 3:**
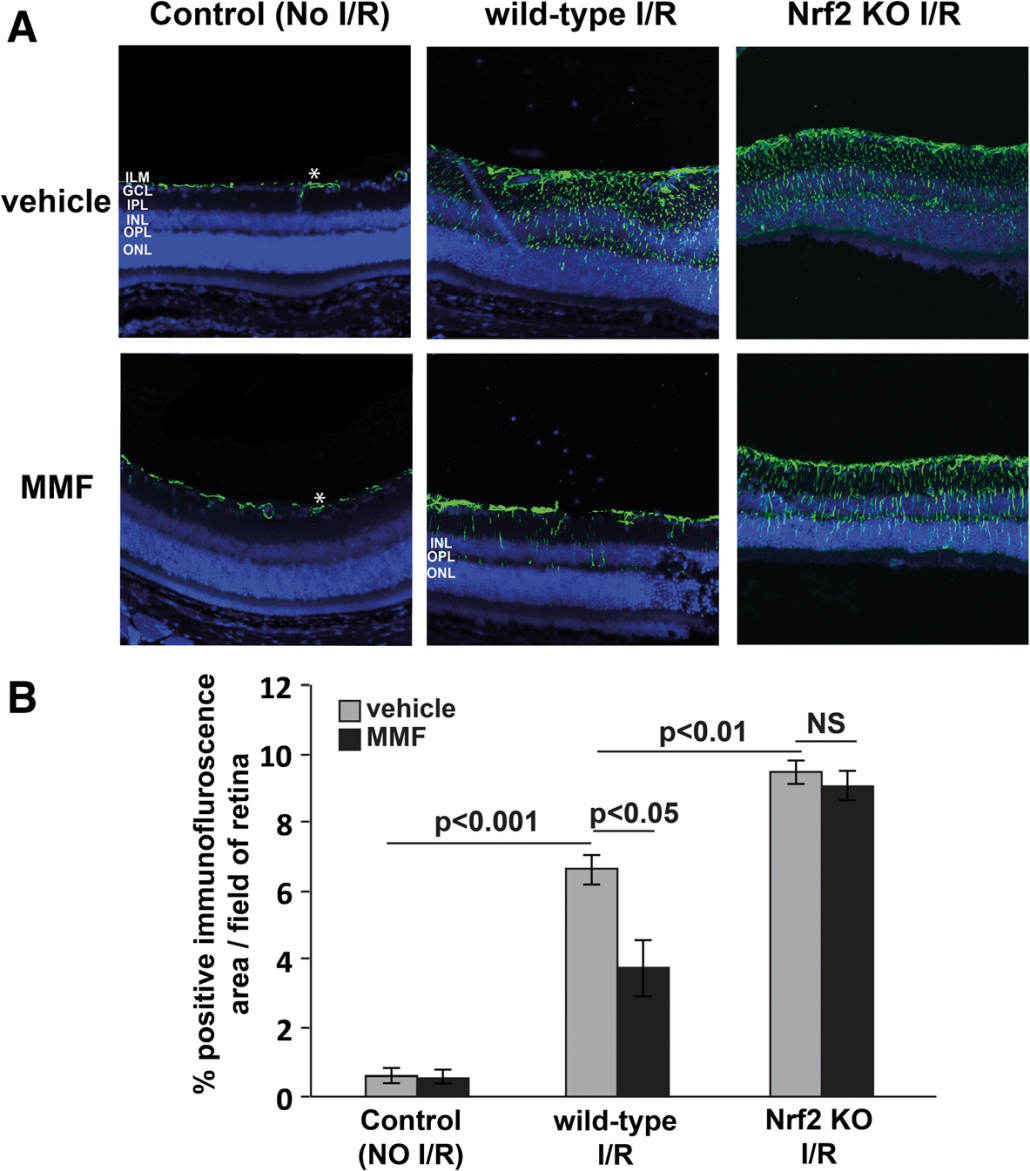
MMF treatment significantly reduces retinal Müller cell gliosis associated with I/R injury at day 7. In control eyes, GFAP immunolabeling was mainly present on the ILM (*asterisk*) (**a**). In wild-type I/R eyes treated with vehicle, the immunolabeling was significantly (*p* < 0.001) increased (**b**) when compared with control eyes and extended almost throughout the entire retina to the ONL in the Müller cell processes (**a**). With MMF injection, the immunolabeling was significantly (*p* < 0.05) reduced (**b**) and mainly extended to the OPL and only a small amount of GFAP immunolabeling was observed in the ONL (**a**). In Nrf2 KO mice, both vehicle- and MMF-treatment showed extensive GFAP immunofluorescence throughout all retina layers to the ONL (**a**). I/R-induced Muller cell gliosis was significantly (*p* < 0.01) higher in Nrf2 KO mice when compared with wild-type mice (**b**). *n* = 4-7 mice/group. *ILM* inner limiting membrane, *GCL* ganglion cell layer, *IPL* inner plexiform layer, *INL* inner nuclear layer, *OPL* outer plexiform layer, *ONL* outer nuclear layer

Retinal ischemia results in reactive gliosis, which is characterized by hypertrophy, proliferation, and upregulated expression of the intermediate filament, including glial fibrillary acidic protein (GFAP), in Müller cells [1, 40–42]. A recent in vitro study suggested that MMF could prevent reactive gliosis by attenuating Müller cell proliferation by decreasing folate uptake via the proton-coupled folate transporter (PCFT) [42]. In order to test if MMF can suppress reactive gliosis in Müller cells in vivo, retinas were collected 7 days after I/R, and GFAP staining was performed. In mammalian retina under non-pathological conditions, GFAP is constitutively expressed in astrocytes but not in mature Müller cells [43]. As observed in Fig. 3a, in control eyes without I/R injury, GFAP immunolabeling was confined to the astrocytes in the ganglion cell layer. With I/R injury, however, GFAP expression was significantly (*p* = 0.0003) upregulated in Müller cells (Fig. 3a, b). MMF treatment significantly (*p* = 0.017) mitigated I/R-induced GFAP expression in the retina as compared with vehicle treatment in the wild-type mice (Fig. 3b). Notably, positive GFAP immunofluorescence spanned all retinal layers from the inner limiting membrane (ILM) to the outer nuclear layer (ONL) in vehicle-treated wild-type I/R eyes, whereas GFAP immunofluorescence was seldom observed in the ONL in MMF-treated eyes. In Nrf2 KO mice, the I/R-induced GFAP upregulation was significantly (*p* = 0.0014) higher as compared with wild-type I/R eyes (Fig. 3b), suggesting that Nrf2 KO mice may be more susceptible to Müller cell gliosis than wild-type mice. As expected, MMF treatment of Nrf2 KO mice did not mitigate the I/R-induced GFAP upregulation; GFAP immunofluorescence spanned all retinal layers regardless of the MMF treatments, implying that the mitigation of GFAP upregulation by MMF is mediated through Nrf2 pathways (Fig. 3a, b).

### MMF treatment protects retinal ganglion cells from ischemia-reperfusion-induced cell death in an Nrf2-dependent manner

**Fig. 4:**
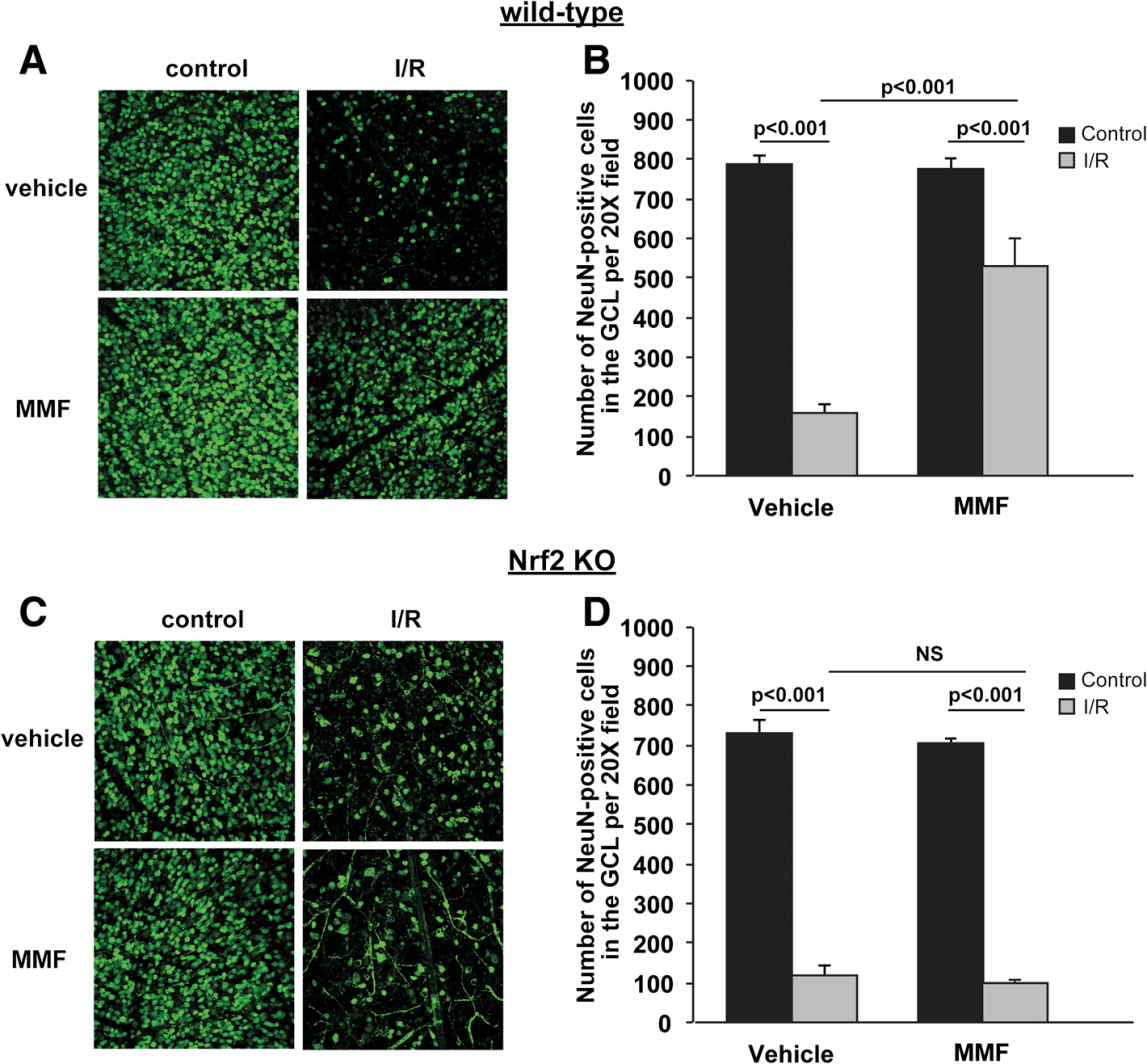
MMF treatment protects retinal ganglion cells from I/R-induced cell death in an Nrf2-dependent manner. Representative anti-NeuN stained retinal flat-mount images from WT (**a**) and KO (**c**) mice are shown. MMF significantly (*p* < 0.001) inhibits neuronal cell loss in GCL at 7 days after I/R in WT (**b**), but not KO (**d**), mice. *n* = 4-6 mice/group

Next, in order to test if the anti-inflammatory effects of MMF are translated into neuronal protection after I/R injury, neuronal cell survival in the ganglion cell layer (GCL) was evaluated at 7 days after I/R by retinal flat-mount analysis with NeuN staining [25, 44] (Fig. 4a, c). Daily i.p. injection of MMF significantly (*p* = 0.0005) increased ganglion cell survival in I/R eyes (33 % cell loss in the MMF-treated group vs. 80 % cell loss in the vehicle-treated group) in wild-type mice (Fig. 4b). Importantly, MMF-mediated inhibition of neuronal cell loss in the GCL was not observed in Nrf2 KO mice (Fig. 4d), indicating that the neuroprotective effect of MMF treatment is mediated by an Nrf2-dependent mechanism.

### MMF inhibits ischemia-reperfusion-induced functional impairment in the retina

**Fig. 5:**
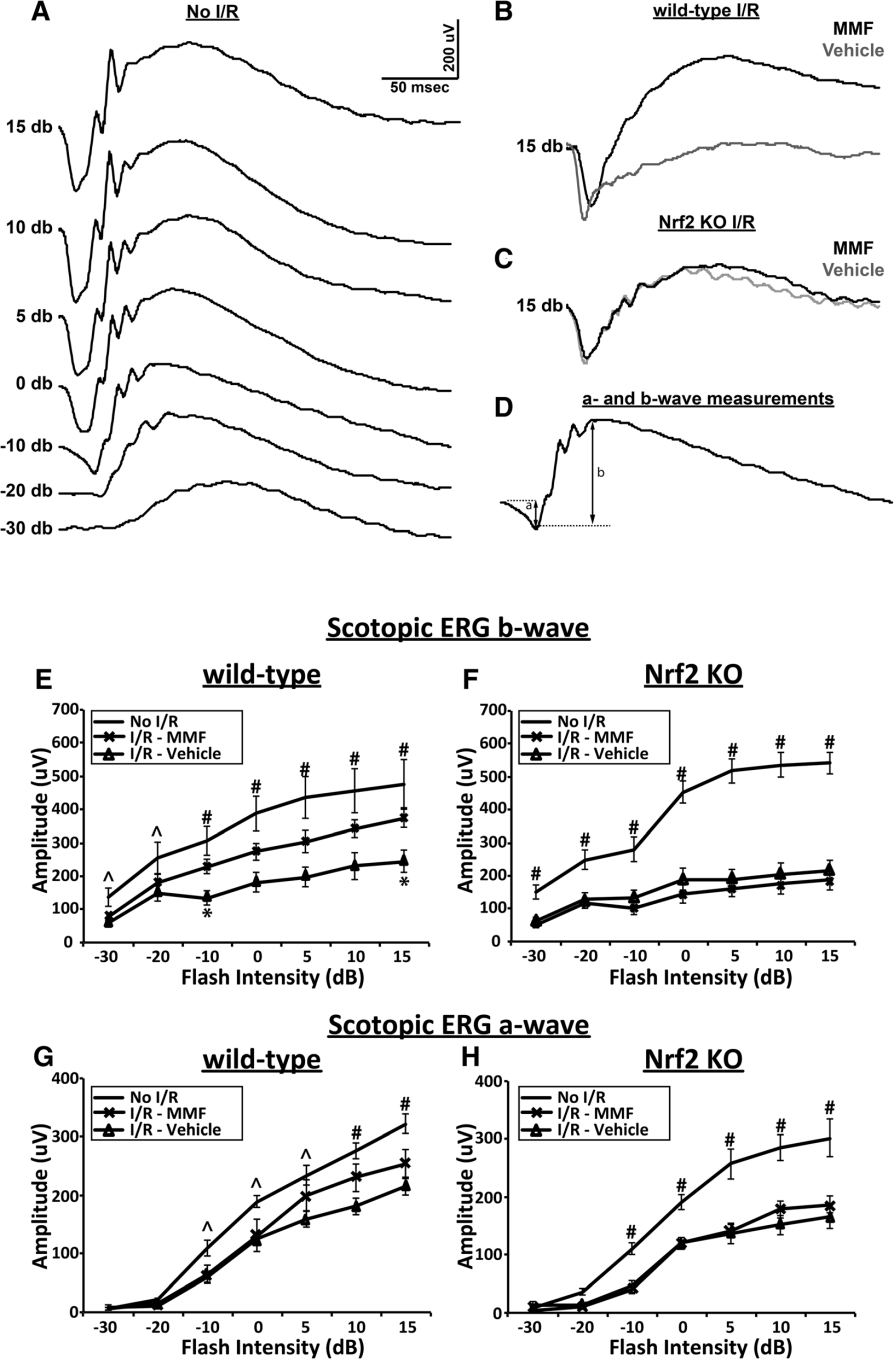
MMF treatment improves I/R-induced functional impairment in the retina in an Nrf2-dependent manner. An intensity series of dark-adapted flash ERG traces from a representative non-I/R eye (**a**), and representative traces from I/R eyes with or without MMF treatment to a stimulus flash of 15 dB in wild-type (**b**) and KO (**c**) mice are shown. An illustration depicting measurements of a and b wave amplitudes are shown (**d**). Retinal I/R injury significantly reduces b wave (**e** and **f**) and a wave (**g** and **h**) amplitudes in both wild-type and KO mice at day 7. With MMF treatment, b wave amplitudes in I/R eyes show significant increases at −10 and 15 dB (**e**), whereas MMF shows no treatment effect in Nrf2 knockout mice (**f**). A non-significant trend toward improved a wave amplitude at higher flash intensities of 5, 10, and 15 dB in wild-type mice after MMF treatment (**g**), whereas no such trend was present in Nrf2 knockout mice (**h**). *p < 0.05, vehicle I/R compared with MMF I/R; ^*p* < 0.05, ^#^ *p* < 0.005, No I/R control compared with vehicle I/R. *n* = 7-10 mice/group

In order to examine the functional changes associated with I/R injury, scotopic ERG was recorded from both WT and Nrf2 KO mice. At 7 days after I/R injury, wild-type mice treated with vehicle showed significant reduction in b wave amplitude at all intensities in I/R eyes as compared with contralateral non-I/R control eyes (top and bottom graphs in Fig. 5e). Comparing vehicle- and MMF-treated I/R eyes (bottom 2 graphs in Fig. 5e), daily i.p. injections of MMF resulted in increased b wave amplitude as compared with vehicle-treated mice. Specifically, MMF treatment resulted in significant increases in b wave amplitude at intensities of −10 dB (*p* = 0.021) and 15 dB (*p* = 0.049) (bottom 2 graphs in Fig. 5e). As anticipated, in Nrf2 KO mice, MMF treatments did not increase b wave amplitude in I/R eyes (bottom 2 graphs in Fig. 5f). There was a non-significant trend toward an improved a wave amplitude at higher flash intensities of 5, 10, and 15 dB in wild-type mice after MMF treatment (Fig. 5g); however, such a trend was not present in Nrf2 KO mice (Fig. 5h).

## Discussion

Recently, fumaric acid esters, specifically DMF and MMF, collectively denoted here as FAEs, have gained considerable attention for their neuroprotective effects in CNS tissues and enhanced survival of various CNS cell types in vitro [8–11, 13]. Although their exact mode of action is not known, the neuroprotective effects of FAEs are thought to be mediated by activation of the Nrf2 pathway [8, 9, 13]. Despite being an extension of the CNS, limited information is available on the therapeutic effects of FAEs on the retina. There have been a few in vitro studies examining the effects of MMF in Müller [42] and retinal pigment epithelial [45, 46] cells, which suggest that the FAEs may have therapeutic potential in retinal pathology.

In this study, we demonstrated using Nrf2 KO mice that FAEs have beneficial effects in vivo on retinal inflammation and neuroprotection in an Nrf2-dependent fashion. Strikingly, there have been only a few in vivo studies that have utilized Nrf2 KO mice to implicate Nrf2 as the critical mechanism of FAEs action [8, 47, 48]. Notably, it has been shown in wild-type but not Nrf2 KO mice that DMF significantly increases axon/myelin preservation with reduction in astrocyte activation in a model of experimental autoimmune encephalomyelitis [8], reduces the size of myocardial infarction in a coronary artery ligation (myocardial I/R) model [47], and improves neurological performance in the model of intracerebral hemorrhage [48]. However, to our knowledge, this is the first in vivo study showing that the effects of FAEs on neuronal cell survival and inflammatory gene expression are mediated by an Nrf2-dependent pathway. Currently, there are no clinically approved neuroprotective drugs for acute and chronic retinal diseases. Therefore, this study highlights FAEs as a promising retinal therapy especially in light of recent FDA approval of DMF for the treatment of multiple sclerosis.

In order to prove that the MMF activates the Nrf2 pathway in the retina, we looked at Nrf2 target gene expression after MMF treatment and demonstrated that MMF indeed upregulates the expression of Nrf2-responsive antioxidative genes in the retina (Fig. 1). In agreement with the upregulation of antioxidative genes, we found significant reduction in inflammatory gene expression after I/R injury in MMF-treated mice (Fig. 2). Importantly, both of these effects were observed in wild-type but not in Nrf2 KO mice, emphasizing the Nrf2 dependence of these beneficial effects of MMF.

Glial functions are altered during pathological processes in the nervous system [49]. Müller cells are the principle glial cells in the neural retina, and they undergo reactive gliosis after acute injury or chronic neuronal stress [50, 51]. We have previously shown that Nrf2 is strongly expressed in both the human and mouse retinas, especially in Müller cells [52], suggesting that pharmacological activation of the Nrf2 pathway in the retina may have a direct impact on Müller cells. Our study showed that the reactive Müller cell gliosis measured by GFAP staining was significantly decreased with MMF treatment (Fig. 3). Interestingly, the extent of Müller cell gliosis in vehicle-treated eyes spanned the entire retina from the inner limiting membrane to the outer nuclear layer, whereas a much smaller degree of gliosis in MMF-treated eyes was mostly limited to the inner retina in the inner plexiform layer and the inner nuclear layer, a pattern very similar to previously reported studies in which oral delivery of antioxidant saffron was employed with retinal stress [53, 54]. This could be due to the fact that the inner and outer retinas demonstrate differential sensitivities to ischemic insult [55, 56]. Because the pressure-induced I/R injury causes a more severe damage to the inner retina, some of the MMFs delivered systemically via intraperitoneal injection might have leaked through the damaged central retinal vessels, making the delivery of drug less effective to the inner retina, while the drug was more effectively delivered to the outer retina via choroidal circulation, which was not affected as strongly by I/R. Nonetheless, I/R eyes treated with MMF showed an overall decrease in reactive Müller cell gliosis when compared with vehicle-treated animals, demonstrating strong neuroprotective effects of MMF in the retina. Contrary to wild-type mice, Müller cell gliosis in Nrf2 KO mice showed no effects from MMF treatment; the gliosis spanned all retinal layers in both MMF- and vehicle-treated groups, implicating that MMF-mediated suppression of Müller cell gliosis is Nrf2-dependent. Because major retinal diseases, including macular degeneration, retinitis pigmentosa, and diabetic retinopathy, are associated with reactive Müller cell gliosis [51], the activation of the Nrf2 pathway by MMF could have a wide implication for the treatment of retinal pathologies.

The neuroprotective effects of MMF in the retina were highlighted by both the significant reduction of neuronal cell death in the ganglion cell layer (Fig. 4) and the recovery of retinal function (Fig. 5). Since ERG b wave amplitude is a particularly sensitive index of retinal ischemia [57] and Müller cells are extensively involved in the generation of b wave [58], we first looked at the b wave of the ERG to see if the MMF-mediated reduction of Müller cell gliosis in I/R eyes is associated with b wave recovery. In accordance with our results from the Müller cell gliosis, we found that the b wave was significantly depressed by the I/R injury and was partially but significantly rescued by the MMF treatment at multiple flash intensities. Although not statistically significant as the b wave recovery, the a wave recovery was also present with the MMF treatment. Taken together, these results imply that the neuroprotective effects of MMF span all retinal layers, since the b wave and a wave reflect the health of neurons in inner and outer layers of the retina, respectively.

In this study, we included the inflammatory gene analyses in addition to the neuroprotective endpoints which were the focus of our study. Although we find these gene studies to be insightful as they indicate that MMF also influences the inflammatory milieu in I/R in an Nrf2-dependent manner, a detailed characterization of response of specific inflammatory cell types will be helpful to gain a further understanding of MMF’s anti-inflammatory effects.

We used MMF because it is the main metabolite that exerts biological effects in vivo after ingestion of DMF [14, 15]. However, recent in vitro studies showed that there are differential effects between DMF and MMF [9, 12, 59]. DMF was shown to be more potent than MMF in the induction of Nrf2 activation in human astrocytes [9] and upregulation of intracellular GSH from oxidative glutamate toxicity in mouse hippocampal cells [12]. In addition, there is a growing evidence that not all DMF pharmacological effects are conveyed by MMF and that DMF may have unique pharmacological properties and thus should not just be considered as a prodrug of MMF [60, 61]. Therefore, further studies using DMF may provide a deeper insight into the action of FAEs in retinal protection.

## Conclusions

Based on the results from concurrent experiments performed in Nrf2 KO mice, we demonstrated that MMF-mediated neuroprotective effects in the retina observed in wild-type mice after I/R injury are indeed Nrf2-dependent. Nrf2 has been known to play a protective role in multiple disease settings [62], and we have previously shown that pharmacological targeting of Nrf2 using synthetic triterpenoids for retinal protection could be a very promising strategy [2, 44]. There are multiple agents that are known to target the Nrf2 pathway, including sulforaphane, VEDA, and synthetic triterpenoids among others. However, these drugs are still in early stages of preclinical or clinical trials [62] or lack human safety data. Therefore, FAEs could be a readily available Nrf2-targeting drug for neuroprotection in the setting of retinal I/R injury or in other conditions for which no treatment options are presently available.

## Additional file

Additional file 1: Table S1. Role of Nrf2 target genes and inflammatory mediators used in this study. (DOCX 101 kb)

## Abbreviations

DMF: dimethyl fumarate
ERG: electroretinogram
FAEs: fumaric acid esters
GCL: ganglion cell layer
I/R: ischemia-reperfusion
ILM: inner limiting membrane
INL: inner nuclear layer
IOP: intraocular pressure
IP: intraperitoneal
IPL: inner plexiform layer
MMF: monomethyl fumarate
ONL: outer nuclear layer
OPL: outer plexiform layer
ROS: reactive oxygen species
