# Supplementary material for: Monomethyl fumarate promotes Nrf2-dependent neuroprotection in retinal ischemia-reperfusion
Source: J Neuroinflammation. 2015 Dec 21;12:239. doi: 10.1186/s12974-015-0452-z (PMC4687295; doi:10.1186/s12974-015-0452-z)
Supplement: Additional file 1: Table S1. — Role of Nrf2 target genes and inflammatory mediators used in this study. (DOCX 101 kb) [file 12974_2015_452_MOESM1_ESM.docx]

Table S1. Role of Nrf2 target genes and inflammatory mediators used in this study

| Gene | Role |
| --- | --- |
| *NQO1* | (NAD(P)H dehydrogenase, quinone 1): Protects cells by preventing redox cycling, which leads to the generation of free radicals and oxidative damage [27]. |
| *HO-1* | (Heme Oxygenase 1): Protects against the cytotoxicity of oxidative stress and apoptotic cell death via its immunomodulatory and anti-inflammatory properties [28]. |
| *Prdx1* | (Peroxiredoxin 1): Reduces hydrogen peroxide and alkyl hydroperoxides and plays an antioxidant protective role in cells |
| *Txnrd1* | (Thioredoxin reductase 1): One of the key players in oxidative stress control. Txnrd1 reduces and activates thioredoxin, which then binds to reactive oxygen species (ROS) before they can harm cells, thus protects cells from oxidative stress [26]. |
| *Gstm1* | (Glutathione S-transferase Mu 1): A member of the GST family enzymes that have broad detoxifying abilities against toxins and oxidative stress by conjugation with antioxidant glutathione (GSH). |
| *IL-1β* | (Interleukin 1 beta) : A proinflammatory mediator known to be associated with variety of neurodegenerative diseases. It has been shown that *IL-1β* plays a pivotal role in mediating ischemic retinal damage [32]. |
| *ICAM-1* | (Intercellular adhesion molecule 1): ICAM-1 mediates endothelium-leukocyte adhesion, which is thought to play a central role in I/R injury. Accumulated leukocytes causes tissue injury by impeding blood flow, or producing superoxide radicals/inflammatory cytokines [33]. |
| *CCL2/CCL7 /CCL12* | (Chemokine (C-C motif) ligand 2, 7, 12): An inflammatory chemokine involved in recruiting and activate/regulate monocytes, macrophages, memory T cells, natural killer cells, and dendritic cells to injury sites through interactions with CCR2 receptor, which is known to play an important role in inflammatory diseases of the CNS [34-37, 39]. |
|  |  |
